# Hybrid periventricular surgery for ventricular septal defects in children: a single-center large-cohort study in Kazakhstan

**DOI:** 10.3389/fcvm.2026.1846675

**Published:** 2026-08-06

**Authors:** Shukhrat Marassulov, Abay Baigenzhin, Akkerbez Adilbekova, Assiya Akhmoldayeva, Saniya Murzabayeva, Timur Raimkhanov, Rinat Maiorov, Yerbol Aldabergenov, Ulan Kabaev, Almas Tolegenuly, Ilkhom Bebezov, Nurlan Tlepov, Oleg Lookin, Xiao Teng, Xiangbin Pan

**Affiliations:** 1 JSC National Scientific Medical Center, Astana, Kazakhstan; 2 Astana Medical University, Astana, Kazakhstan; 3 International Higher School of Medicine, Bishkek, Kyrgyzstan; 4 Medical Center for Cardiology and Internal Medicine “Archimed”, Aktau, Kazakhstan; 5 National Center for Cardiovascular Diseases, Chinese Academy of Medical Sciences and Peking Union Medical College, Beijing, China

**Keywords:** cardiopulmonary bypass, complete atrioventricular block, hybrid surgery, late rhythm disturbance, mini-invasive surgery, pediatric patients, transient rhythm disturbance, ventricular septal defect

## Abstract

**Background:**

The hybrid technique for ventricular septal defect (VSD) closure combines elements of conventional open-heart surgery with cardiopulmonary bypass (CPB) and minimally invasive approaches, including beating-heart occluder implantation through right ventricular free-wall puncture. Hybrid periventricular VSD closure offers several advantages over CPB-based surgery. Its less invasive nature, facilitated by catheter-based access to the defect, may reduce anesthesia duration and hospital stay in pediatric patients. Despite these potential benefits, the adoption of hybrid VSD correction remains limited, particularly in Kazakhstan.

**Methods:**

We report our experience with hybrid VSD closure in 747 pediatric patients (age range: 0–18 years) treated between 2016 and 2024, representing the first implementation of this technique in Kazakhstan. We evaluated the impact of pre-, peri-, and postoperative variables, including VSD size and type, occluder characteristics, duration of anesthesia, and mechanical ventilation, on the risk of early or late rhythm disturbances and the need for conversion to CPB.

**Results:**

Postoperative complications occurred in 27 of 747 patients. Seven patients developed early transient rhythm disturbances, 10 experienced complete atrioventricular block (cAVB) between 2 weeks and 3.5 years after surgery, and 8 required conversion to CPB. Early transient rhythm disturbances were not associated with any analyzed factors. In contrast, late cAVB was significantly associated with older age (>5 years) and larger VSD size (>8 mm). Inlet VSD morphology, larger VSD size, and their combination were significant predictors of CPB conversion. Based on these findings, we propose an algorithm for patient selection for minimally invasive hybrid VSD closure.

**Conclusions:**

Several clinical and anatomical factors should be considered when assessing perioperative and long-term risks of hybrid VSD closure in pediatric patients. Nevertheless, the hybrid approach represents a valuable alternative to conventional surgery, demonstrating a low incidence of long-term complications.

## 1 Introduction

Congenital heart defects (CHDs) comprise a broad spectrum of structural cardiac abnormalities and remain one of the leading causes of morbidity and mortality in the pediatric population. Many CHDs are life-threatening and require early intervention, often during the neonatal period and, in selected cases, even before birth (1, 2). The reported incidence of CHDs ranges from 4 to 10 per 1,000 live births, although earlier studies have reported substantially higher estimates of up to 50 per 1,000 live births (3, 4). Ventricular septal defects (VSDs) are among the most common congenital heart defects, accounting for approximately 25%–40% of all CHDs, with an estimated incidence of approximately 4 per 1,000 live births (4–8). Depending on their size and anatomical location, persistent VSDs may result in heart failure, pulmonary hypertension, impaired somatic growth, and increased mortality, thereby necessitating timely intervention.

Several treatment options are currently available for VSD closure, including conventional open-heart surgery, transcatheter device closure, and hybrid techniques that combine surgical and interventional approaches. Conventional surgical repair remains the standard treatment for many VSD subtypes; however, it requires cardiopulmonary bypass (CPB), which is associated with procedure-related morbidity, particularly in infants and young children. The principal concerns include neurological complications related to CPB and anesthesia, systemic inflammatory responses, and coagulation disorders (9–11). Furthermore, conventional open-heart surgery is associated with longer operative times, prolonged intensive care unit (ICU) stay, and extended postoperative hospitalization compared with less invasive approaches (12).

Minimally invasive surgical techniques, including mini-thoracotomy and mini-sternotomy, represent less traumatic alternatives to conventional CPB-assisted surgery while preserving the advantages of direct surgical repair (13). For example, right lateral and right anterior mini-thoracotomy have been successfully used for VSD closure, resulting in shorter CPB duration than conventional median sternotomy (14, 15). Completely CPB-free transthoracic device closure was introduced more than a decade ago; however, its use remains limited in very small children (16, 17). In addition, delayed complete atrioventricular block (cAVB) has been reported following this procedure (18). Transcatheter device closure is another widely adopted technique with high procedural success rates; however, it is associated with a relatively high risk of unplanned reintervention (19). Moreover, postprocedural arrhythmias of various origins have been reported in up to approximately 25% of children undergoing transcatheter closure of perimembranous VSDs (20). A recent multicenter study involving more than 3,000 pediatric patients who underwent minimally invasive thoracotomy between 1999 and 2024 demonstrated that this approach is both safe and effective (21). Nevertheless, the authors reported an overall complication rate of approximately 1.5%, including wound infections, neurological complications, and clinically significant rhythm disturbances, as well as a reoperation rate of 0.73%.

The earliest hybrid approaches to VSD closure still required CPB support (22). However, contemporary hybrid periventricular VSD closure has evolved into a completely CPB-free technique for appropriately selected patients, particularly low-weight infants and children with muscular VSDs. Although clinical experience with this approach has increased substantially over the past decade, comprehensive evidence regarding perioperative outcomes and long-term follow-up remains limited. Therefore, the present study aimed to evaluate both early clinical outcomes and long-term results, with follow-up extending to 9 years, in pediatric patients undergoing hybrid periventricular VSD closure at our institution.

## 2 Materials and methods

### 2.1 Study population

We retrospectively analyzed the clinical data of 747 pediatric patients aged 2 months to 17.8 years who underwent ventricular septal defect (VSD) closure using a hybrid periventricular beating-heart approach between 2016 and 2024 at a single tertiary cardiac center. All procedures were performed under normal central hemodynamic conditions without the routine use of cardiopulmonary bypass (CPB). Patients were considered eligible for hybrid VSD closure if they met the following criteria:

- isolated muscular or perimembranous VSD;
- favorable VSD anatomy suitable for hybrid closure, including an aortic rim of at least 2 mm;
- feasibility of intraoperative transesophageal echocardiography (TEE);
- body weight ≥3.5 kg.

### 2.2 Preoperative assessment

All patients underwent a standardized preoperative evaluation that included a 12-lead electrocardiogram (ECG), chest radiography, and comprehensive transthoracic echocardiography (TTE). Standard subcostal, apical, and parasternal echocardiographic views were used to assess defect morphology. Morphological assessment included determination of the VSD location, left- and right-sided defect diameters, defect length, and measurements of the surrounding anatomical margins, including the subaortic margin (distance between the aortic valve annulus and the superior margin of the defect) and the tricuspid margin (distance between the tricuspid valve annulus and the inferior margin of the defect).

### 2.3 Occluder selection

The final occluder size was selected intraoperatively based on detailed TEE measurements, including VSD type, left- and right-sided orifice diameters, presence of aneurysmal tissue, and dimensions of the subaortic and tricuspid rims. For perimembranous VSDs with an adequate subaortic rim, the device diameter was generally selected to be 2 mm larger than the maximum diameter of the left ventricular orifice. When there was concern regarding possible contact between the device and the aortic valve, the occluder size was reduced to correspond to the left ventricular diameter of the defect. In the presence of a large aneurysm, the device was positioned within the aneurysmal pouch rather than at the entrance of the left ventricular defect. For muscular VSDs, device selection followed similar principles. The left ventricular disc was selected to exceed the maximal defect diameter by approximately 2 mm.

### 2.4 Occluder devices

Two commercially available occluder systems were used throughout the study: LifeTech and Lepu Medical (China). Both devices are self-expanding, low-profile nitinol occluders consisting of two discs with an embedded polytetrafluoroethylene membrane. Appropriate delivery systems supplied by the manufacturers were used for device implantation.

### 2.5 Surgical technique

All procedures were performed under general anesthesia with continuous TEE guidance. Patients received intravenous heparin (70 IU/kg) immediately before device implantation together with prophylactic cefazolin (50 mg/kg), followed by two additional postoperative doses. Surgical access was obtained through a 2–3 cm right mini-thoracotomy. After opening the pericardium, the avascular region of the anterior right ventricular wall was identified, and a purse-string suture was placed. After confirming the VSD dimensions by TEE, the appropriate occluder was selected, typically 1–2 mm larger than the maximal VSD diameter. A guidewire was advanced across the defect, followed by insertion of the delivery sheath into the left ventricle. The left ventricular disc was deployed first, followed by gradual deployment of the right ventricular disc. Correct device positioning, complete defect closure, valve competence, and hemodynamic stability were verified using intraoperative TEE. If satisfactory device position and complete VSD closure were confirmed without valve dysfunction or significant residual shunting, the delivery system was removed and the patient was transferred to the intensive care unit. Postoperatively, heparin (70 IU/kg) was administered every 4–6 h during the first 24 h after surgery.

### 2.6 Follow-up

Immediate postoperative outcomes included assessment of: a) procedural success; b) hemodynamic parameters; c) duration of mechanical ventilation; d) intensive care unit stay; e) total hospital stay; f) postoperative complications. Long-term outcomes were evaluated using clinical examinations and echocardiography performed at 1, 3, 5, and 9 years after hybrid VSD closure.

### 2.7 Ethical approval

Written informed consent for hybrid VSD closure was obtained from the parents or legal guardians of all patients before surgery. Because the present investigation was designed as a retrospective study initiated in 2023, additional consent for publication of anonymized clinical data was not required. All data were fully de-identified before analysis. The study was conducted in accordance with the principles of the Declaration of Helsinki and was approved by the Local Ethics Committee of JSC National Scientific Medical Center (Protocol No. 053/CT-75, October 27, 2023).

### 2.8 Statistical analysis

Statistical analyses were performed using Statistica 10 and GraphPad Prism 7. Continuous variables are presented as the median and interquartile range (IQR). Between-group comparisons were performed using the Kruskal–Wallis test followed by Dunn's multiple-comparison test. Paired data were analyzed using the Wilcoxon matched-pairs signed-rank test. Categorical variables were compared using the chi-square test. The association between occluder characteristics and residual shunt was evaluated using Spearman's rank correlation coefficient. To identify independent and interactive predictors of early and late postoperative complications, including rhythm disturbances and conversion to CPB, factorial analysis of variance (ANOVA) was performed. A two-sided *p*-value <0.05 was considered statistically significant.

## 3 Results

### 3.1 Patient characteristics

The baseline demographic and clinical characteristics of the study population, together with the anatomical features of the ventricular septal defects (VSDs) and the implanted occluders, are summarized in Table 1. Of the 747 patients included in the study, 410 (54.9%) were female. The largest age subgroup comprised children 1–5 years of age (331 patients, 44.3%). Overall, children aged ≤5 years accounted for more than 65% of the study population, resulting in a markedly skewed age distribution. Patient age ranged from 2 months to 17.8 years. Body weight ranged from 3.5 to 75 kg, with a median of 13.5 kg (IQR: 9.2–21.1 kg). More than half of the patients (428, 57.3%) weighed between 10 and 30 kg at the time of surgery. At least one concomitant medical condition was present in 226 patients (30.3%).

**Table 1:** Data of patients operated between 2016 and 2024 (*n* = 747) and characteristics of their VSD/occluder used.

Parameter	Range or type of parameter	Value or number of patients (%)
Gender	Male	337 (45.1%)
	Female	410 (54.9%)
Median age (IQR), years		2.8 (1.1–6.6)
Age range	<1 year	161 (21.6%)
	1–5 years	331 (44.3%)
	6–11 years	177 (23.7%)
	12–18 years	78 (10.4%)
Median weight (IQR), kg		13.5 (9.2–21.1)
Weight range	Less than 10 kg	216 (28.9%)
	From 10 to 30 kg	428 (57.3%)
	More than 30 kg	103 (13.8%)
Comorbidity	Yes	226 (30.3%)
	No	521 (69.7%)
VSD type	Perimembraneous	549 (73.7%)
	Muscular	86 (11.5%)
	Inlet	26 (3.5%)
	Lack of aortic edge	68 (9.1%)
	Recanalization	16 (2.1%)
Median size of VSD (IQR), mm		5.0 (5.0–7.0)
Range of VSD size	≤5 mm	436 (58.4%)
	>5 … ≤9 mm	290 (38.8%)
	>9 mm	21 (2.8%)
Median occluder size (IQR), mm		7.0 (6.0–8.0)
Range of occluder size	≤6 mm	357 (47.7%)
	>6 … ≤10 mm	368 (49.2%)
	>10 mm	23 (3.1%)
Occluder type	Symmetric	259 (34.7%)
	Muscular	256 (34.3%)
	Eccentric	211 (28.2%)
	Assymetric	21 (2.8%)
Occluder brand	LifeTech (Cera)	418 (56.0%)
	Lepu (MemoPart)	328 (43.9%)
	Not indicated in medical history	1 (0.1%)

IQR, interquartile range; VSD, ventricular septal defect.

Perimembranous VSD was the predominant defect type, accounting for 549 cases (73.7%), followed by muscular VSD (86 patients, 11.5%) and VSD associated with the absence of an aortic rim (68 patients, 9.1%). Less common defect types included inlet VSD (26 patients) and recanalization (16 patients), together representing 5.6% of the cohort. The median VSD diameter was 5 mm (IQR: 5.0–7.0 mm). More than half of the patients (436, 58.4%) had defects measuring ≤5 mm. Only 21 patients (2.8%) presented with defects larger than 9 mm, including one patient each with defects measuring 14 mm and 18 mm, three patients with 12-mm defects, and six patients with 11-mm defects. No significant correlation was observed between VSD size and patient age. The median occluder-to-VSD size ratio was 1.2 (IQR: 1.0–1.4), consistent with our institutional protocol for device selection.

### 3.2 Occluder characteristics

**Figure 1:**
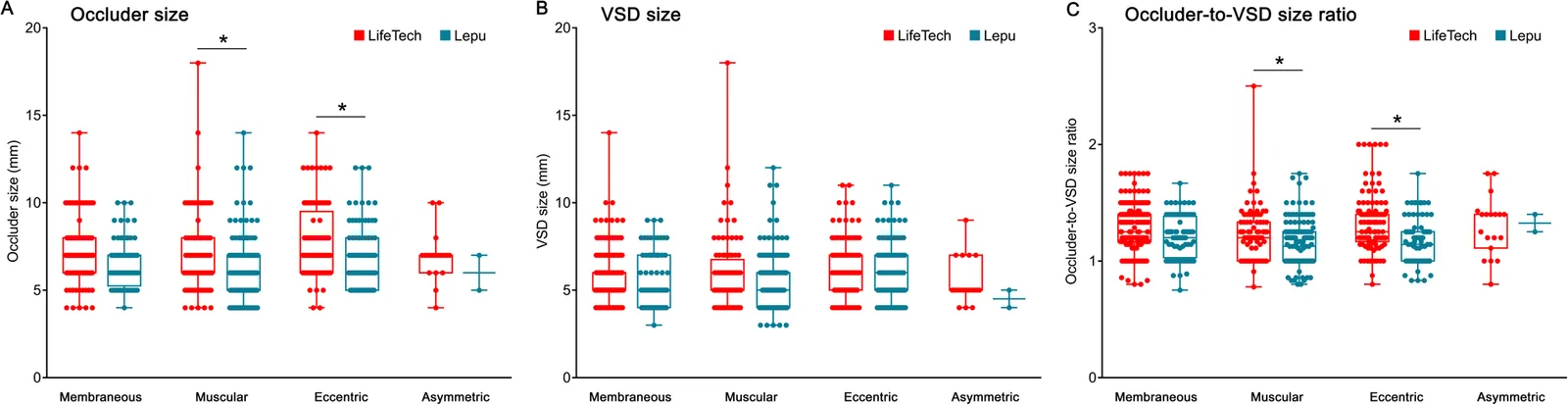
Occluder size (panel **A**), VSD size (panel **B**), and occluder-to-VSD size ratio (panel C) for two occluder brands—LifeTech and Lepu—used in this study. The plots show significant difference between brands, if indicated by asterisk, *p* < 0.01 for all comparisons except in panel C for muscular type of occluder (*p* = 0.0425).

Among the implanted devices, LifeTech/Cera occluders were used in 418 patients (56.0%), whereas Lepu/MemoPart occluders were implanted in 328 patients (43.9%). The manufacturer was not documented for one patient. The distribution of occluder designs was relatively balanced: 259 symmetric devices (34.7%), 256 muscular devices (34.3%), 211 eccentric devices (28.2%), and 21 asymmetric devices (2.8%). We next evaluated whether occluder brand or device design influenced occluder size, VSD size, or the occluder-to-VSD size ratio. For both muscular and eccentric occluders, the mean implanted device diameter was significantly greater for LifeTech than for Lepu devices (*p* = 0.0004 and *p* = 0.0032, respectively; Figure 1A). Likewise, the occluder-to-VSD size ratio was significantly higher for LifeTech devices than for Lepu devices (*p* < 0.0001 and *p* = 0.0425, respectively; Figure 1C). In contrast, the mean VSD diameter did not differ significantly between patients receiving LifeTech and Lepu devices within the same occluder category (Figure 1B), indicating that device manufacturer was not selected according to defect size.

These differences most likely reflect procedural refinements introduced during the study period rather than intrinsic differences between manufacturers. Initially, our institutional protocol recommended selecting an occluder 2 mm larger than the measured VSD diameter. As our experience increased, this strategy was modified to favor devices approximately 1 mm larger than the defect. In addition, fluctuations in device availability resulted in alternating use of the two manufacturers over the study period. Consequently, the observed differences between LifeTech and Lepu devices should not be interpreted as evidence of superior performance of either manufacturer.

### 3.3 Residual shunt

**Figure 2:**
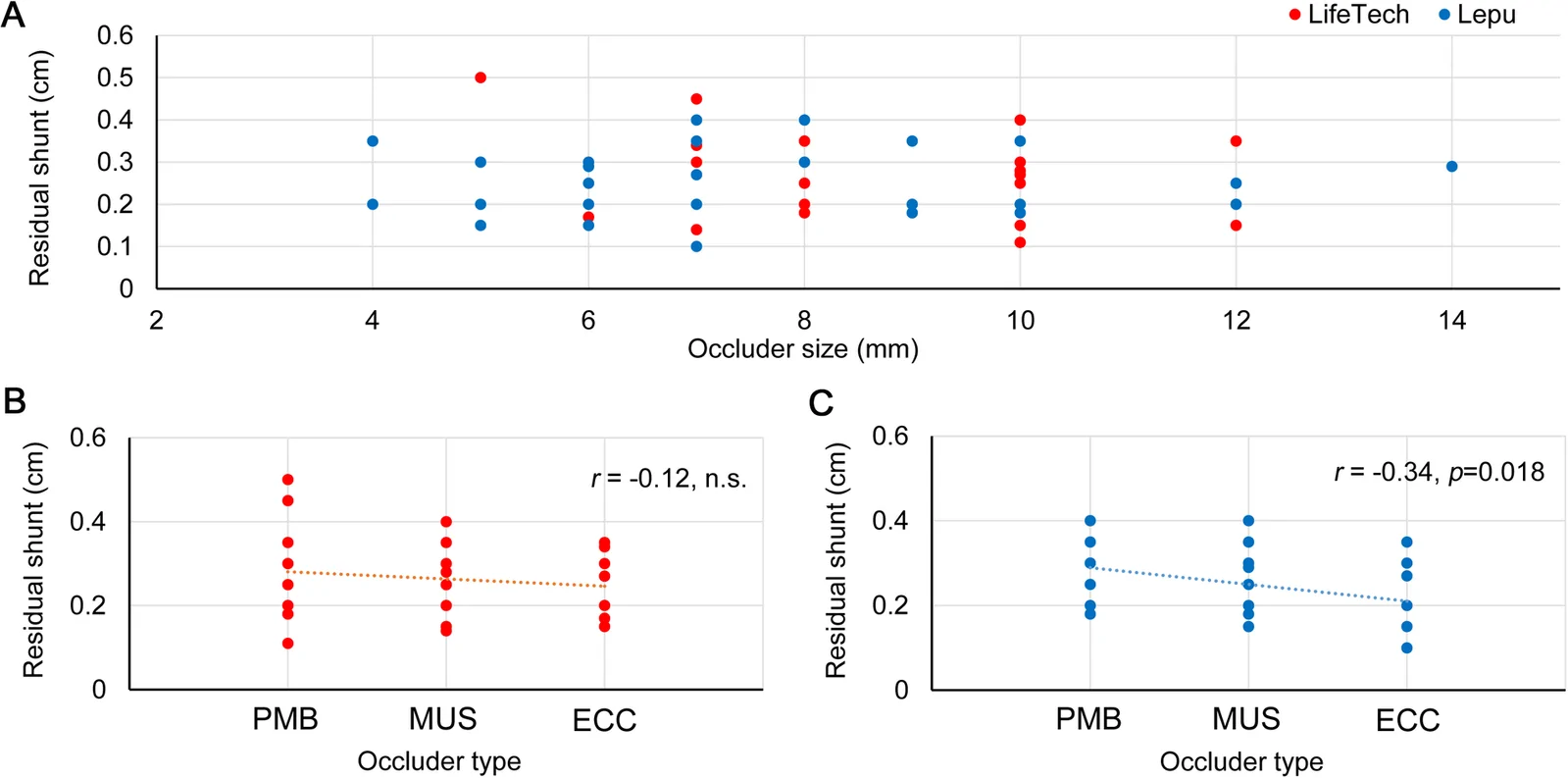
The brand-related effect of occluder size or occluder type on the occurrence of residual shunt. Occluder size vs. residual shunt (panel **A**) and occluder type vs. residual shunt (panels **B,C**) are shown for two occluder brands—LifeTech and Lepu—used in this study. The plot on panel **A** shows no significant difference between brands. The plots on panels **B,C** show that occluder type (PMB, perimembraneous; MUS, muscular; ECC, eccentric) has significant effect on the amount of residual shunt only for Lepu devices (panel **C**, *p* = 0.018) but not for LifeTech devices (panel **B**, *p* = 0.487). Dotted lines in panels **B,C** show linear regression line, with corresponding Spearman *r* value indicated.

Occluder size was not associated with the occurrence of residual shunting, either in the pooled cohort or when LifeTech and Lepu devices were analyzed separately (Figure 2). When residual shunting was analyzed according to occluder type, a significant association was observed among Lepu devices (Figure 2B, right panel). Perimembranous occluders demonstrated the highest incidence of residual shunting, muscular devices showed an intermediate incidence, and eccentric devices demonstrated the lowest incidence. Although the same trend was observed for LifeTech devices, the association did not reach statistical significance. Analysis of the pooled dataset confirmed a significant relationship between occluder design and residual shunting (Spearman's *ρ* = −0.23, *p* = 0.037, not plotted).

### 3.4 Early postoperative outcomes

Preoperative left ventricular ejection fraction (LVEF) was available for 696 patients (93.2%) and had a median value of 67% (IQR: 65%–71%). Early postoperative LVEF was measured in 700 patients (93.7%), with a median value of 65% (IQR: 60%–66%). Among the 686 patients with both measurements available, postoperative LVEF decreased modestly but significantly compared with baseline (Wilcoxon matched-pairs signed-rank test, *p* < 0.0001). The median reduction was 2% (IQR: 0%–7%), with the largest observed decrease of 28% and the largest increase of 26%. The interventricular gradient was measured in 644 patients (86.2%), its median value was 60 mmHg (IQR: 50–70), with a minimum of 6 mmHg and a maximum of 118 mmHg. Residual shunting was detected in 85 patients (11.4%), with a median shunt diameter of 0.2 cm (IQR: 0.2–0.3 cm; range: 0.1–0.5 cm). The median surgical wound length was 3 cm (IQR: 3.0–3.5 cm). Wound length was ≤3 cm in 533 patients (74.8%), whereas 172 patients (24.1%) had an incision measuring 3–5 cm. Only eight patients (1.1%) required an incision exceeding 5 cm, all of whom underwent conversion to conventional surgery with cardiopulmonary bypass.

### 3.5 Immediate procedural outcomes

The immediate efficacy and safety outcomes of hybrid VSD closure are summarized in Table 2. Successful device implantation without major hemodynamic compromise or persistent complications was achieved in 727 patients (97.3%). Among these patients, seven developed transient rhythm disturbances that resolved completely before hospital discharge. The median duration of postoperative mechanical ventilation was 4.5 h (IQR: 3.5–5.5 h). Most patients (583, 78.1%) required ventilatory support for <6 h, whereas 136 patients (18.2%) required ventilation for 6–12 h. Prolonged mechanical ventilation (>12 h) was necessary in 28 patients (3.7%), including three patients who required ventilatory support for 15 days. One of these patients died as a consequence of severe infectious complications. The median duration of intensive care unit stay was 24 h (IQR: 22–24 h), whereas the median postoperative hospital stay was 7 days (IQR: 6–8 days). Cardiotonic agents were not required in 666 patients (89.2%). The remaining 81 patients (10.8%) received dopamine, dobutamine, epinephrine, or their combinations. Monotherapy was sufficient in 68 patients, whereas 13 patients required two or more inotropic agents. One patient required triple-agent inotropic support and subsequently underwent conversion to CPB. Immediately after device deployment, 662 patients (88.6%) had complete defect closure without residual shunting, whereas 85 patients (11.4%) had a hemodynamically non-significant residual shunt ranging from 0.1 to 0.5 cm.

**Table 2:** Immediate results of VSD correction using a hybrid method.

Parameter	Value or number of patients (%)	Remarks
Number of patients without conversions or delayed complications	727 (97.3%)	In 7 patients, transitory rhythm disturbances were successfully resolved
Median duration of mechanical respiratory support (IRQ), h	4.5 (3.5–5.5)	More than 24 h—11 patients, 15 days or more—3 patients, max. duration—24 days (1 patient)
Median ICU stay for the entire patient cohort (IQR), h	24 (22–24)	More than 72 h—10 patients, max. duration—528 h (1 patient)
Median postoperative stay for the entire patient cohort (IQR), days	7 (6–8)	More than 14 days—40 patients, max. duration—47 days (1 patient)
Number of patients with hemodynamically non-significant residual shunt	662 (88.6%)	No residual shunt
	85 (11.4%)	With residual shunt
Number of patients without the need for using cardiotonic drugs	666 (89.2%)	—
Number of patients with the need for using cardiotonic drugs	81 (10.8%)	—
Those required one drug	68 (9.1%)	5 patients had complications or were converted to CPB
Those required two drugs	12 (1.6%)	2 patients had peri-operative or early post-operative sinus bradycardia or cAVB
Those required three drugs	1 (0.1%)	The patient was converted to CPB
Number of patients with peri-operative or delayed complications, including:	21 (2.8%)	—
Conversion to CPB	11 (1.5%)	Indications for the technique have not been strictly followed
Occluder dislocation	3 (0.4%)	The selection of improper occluder
Heart rhythm disturbance	2 (0.27%)	Large VSD in patients under 1 year of age
Increasing aortic regurgitation	2 (0.27%)	Patients had no aortic edge
In-hospital lethality	2 (0.27%)	Due to infectious complications

IQR, interquartile range; ICU, intensive care unit; CPB, cardio-pulmonary bypass; VSD, ventricular septal defect.

### 3.6 Post-surgical complications

A total of 28 patients (3.7%) developed transient or persistent complications. In 21 patients (2.8%), delayed persistent atrioventricular block or supraventricular tachycardia, as well as suspected occluder dislocation associated with a significant residual shunt, were detected either during the procedure or within the first hours after occluder implantation during scheduled control echocardiography. In 8 of these 21 patients, conversion to cardiopulmonary bypass (CPB) was performed, followed by surgical VSD repair using the conventional approach. All patients with perioperative or early postoperative complications were discharged from the hospital in satisfactory clinical condition. The types of complications observed in these 21 patients are presented in Table 2.

In-hospital mortality occurred in two cases (0.27% of the total cohort) and was associated with postoperative infectious complications. One patient died 23 days after treatment due to COVID-19 pneumonia (in 2020), and another patient died 16 days after treatment due to multiple organ failure secondary to infectious complications. Both patients had severe preoperative comorbidities: stage 3 arterial hypertension with very high cardiovascular risk (one patient) and secondary atrial septal defect, Down syndrome (simple form), and grade 3 pulmonary hypertension with congestive heart failure (the other patient). Therefore, no fatal outcomes were directly associated with the hybrid VSD correction technique itself.

### 3.7 Follow-up outcomes

Long-term outcomes were assessed at 1, 3, 5, and 9 years after the procedure using questionnaires, scheduled echocardiographic examinations, and clinical evaluations by physicians. Of the 747 operated patients, 712 (95.3%) were included in the long-term follow-up analysis. All 712 patients completed the 1-year follow-up assessment. Among these patients, 565 (79.4%) completed the 3-year follow-up, 309 (43.4%) completed the 5-year follow-up, and 39 (5.5%) completed the 9-year follow-up. No deaths were recorded after hospital discharge, including during the longest follow-up period.

**Figure 3:**
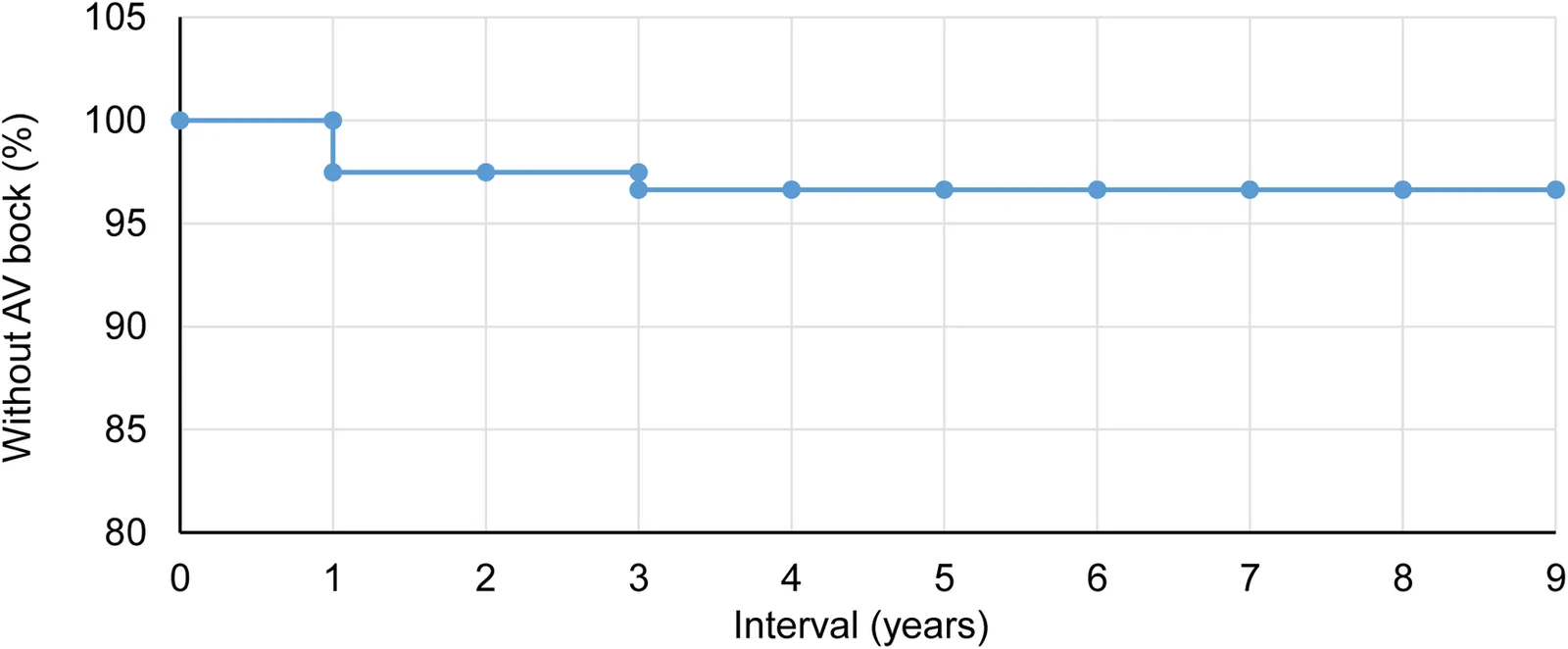
Kaplan–Meier analysis of the incidence of late complete atrioventricular block (cAVB) requiring cardiac pacemaker implantation during long-term follow-up. The observation period extended to 9 years. The *Y*-axis is truncated and begins at 80%.

During long-term follow-up (more than two years after the procedure), two children required repeat surgery due to progressive aortic valve insufficiency caused by partial compression of an aortic valve leaflet by the occluder disk. In one patient, the affected leaflet was successfully dissected and preserved, whereas in the second patient, the damaged leaflet was excised and reconstructed using an autologous pericardial patch. Both children remain under outpatient follow-up. The most frequent late complication was complete atrioventricular block (cAVB). Permanent cardiac pacemaker implantation was required in 10 patients. The timing of pacemaker implantation after hybrid VSD correction was as follows: two weeks after surgery in 2 patients (aged 1 and 4 years at the time of VSD correction); two months after surgery in 1 patient (aged 2 months); six months after surgery in 3 patients (1 patient aged 6 months and 2 patients aged 2 years); one year after surgery in 2 patients (aged 2 years); two years after surgery in 1 patient (aged 1 year and 2 months); and 3.5 years after surgery in 1 patient (aged 10 months). Figure 3 demonstrates the Kaplan–Meier curve illustrating the annual incidence of late cAVB requiring permanent cardiac device implantation. The curve indicates that cAVB occurred exclusively within the first 3–4 years after hybrid VSD correction. No new cases of this complication were identified during extended follow-up periods of 5 and 9 years after VSD correction.

Table 3 summarizes the clinical characteristics of patients who developed late cAVB. Three male and seven female patients aged between 6 months and 13.6 years underwent hybrid VSD correction between July 2019 and August 2021. All patients were diagnosed with perimembranous VSD. One patient had a concomitant absence of the aortic rim. Before corrective surgery, all patients were classified as NYHA functional class III for heart failure. Two patients had significant comorbidities: one patient had pre-existing rhythm and conduction abnormalities, including second-degree atrioventricular block (Mobitz II) and transient cAVB, while another patient had severe pulmonary hypertension. The VSD size ranged from 4 to 12 mm, with 50% of patients having a defect size of 5 mm. The occluder diameter ranged from 6 to 14 mm. The median blood loss was 10 mL, with a minimum of 5 mL (two patients) and a maximum of 50 mL (one patient).

**Table 3:** The list of patients who suffered from permanent heart rhythm disturbances that required the implantation of cardiac pacemakers (subgroup B).

#	G-r	Age (yr)	*W* (kg)	*H* (cm)	Diagnosis	Comorbidity	Occ/VSD size (mm)	Occ/VSD ratio	Occ type	Blood loss (mL)	Anesth/VS (h)	Wound size (cm)	LVEF bef/aft (%)	IVPG (mmHg)
1	F	1.3	8.7	82	pmVSD, no aortic edge, HF NYHA class III	No	10/5	2.00	ECC	50	2/4	4	70/68	80
2	М	0.8	9.6	74	pmVSD, HF NYHA class III	No	7/5	1.40	ECC	5	2/5.5	3	69/69	63
3	F	1.2	9	74	pmVSD, HF NYHA class III	No	6/5	1.20	ECC	30	2/5	3	61/61	50
4	М	0.8	10	81	pmVSD, HF NYHA class III	No	8/7	1.14	ECC	5	2/5.5	3	—/65	—
5	М	13.6	50	152	pmVSD, HF NYHA class III	Rhythm and conduction disturbance, 2nd degree AVB (MOBITZ II), transient cAVB	14/12	1.17	Membr	25	3/6	5	65/60	80
6	F	0.5	6.2	63	pmVSD, no aortic edge, HF NYHA class III	High pulmonary hypertension	10/7	1.43	Asymm	10	2/4.5	3	65/60	60
7	F	2.3	10.5	89	pmVSD, HF NYHA class III	No	6/5	1.20	Membr	10	2/5	3	76/65	90
8	F	0.8	7.4	70	pmVSD, HF NYHA class III	No	12/10	1.20	ECC	10	2/6.5	3	—/59	—
9	F	1.1	11	81	pmVSD, HF NYHA class III	No	6/5	1.20	ECC	10	2/2.5	3	70/64	70
10	F	1.7	12	78	pmVSD, HF NYHA class III	No	6/4	1.50	ECC	10	2/3.5	3	60/60	—

Abbreviations in the column titles are as follows: G-r, gender; yr, years; W, weight; H, height; Occ, occluder; VSD, ventricular septal defect; pmVSD, perimembraneous VSD; Anesth, anesthesia (operative); VS, ventilation support; h, hours; LVEF, left ventricular ejection fraction; bef, before; aft, after; IVPG, inter-ventricular pressure gradient; HF, heart failure; cAVB, complete atrio-ventricular block; ecc, eccentric type of occluder; membr, membraneous type of occluder; asymm, asymmetric type of occluder. “–” indicates no available data. In all these patients we used LifeTech occluder device.

### 3.8 Risk factors for late complications

To identify factors associated with delayed complications, we performed separate frequency analyses for patients who developed one of the following outcomes: transient rhythm disturbances that resolved before hospital discharge (subgroup A, *n* = 7), late cAVB requiring implantation of a permanent cardiac pacemaker after hospital discharge (subgroup B, *n* = 10), and inappropriate occluder implantation requiring conversion to CPB (subgroup C, *n* = 8). The reference group consisted of patients from the main cohort without any complications (*n* = 720).

The duration of anesthesia, duration of respiratory support, and incision size did not differ significantly between complication-free patients (*n* = 720) and patients with late cAVB (subgroup B; *p* > 0.145 for all parameters). Similarly, neither anesthesia duration nor incision size was associated with the risk of transient rhythm disturbances (subgroup A; *p* > 0.5 for all parameters compared with the reference cohort). Anesthesia duration was also comparable among all subgroups, indicating that it was not a determinant of the type of complication (*p* > 0.5). In contrast, the duration of respiratory support was associated with an increased incidence of transient rhythm disturbances (subgroup A, respiratory support lasting 6–12 h; *p* = 0.004 vs. reference cohort) and conversion to CPB (subgroup C, respiratory support lasting >12 h; *p* < 0.001 vs. reference cohort). An incision size >5 cm was significantly more frequent in subgroup C compared with the reference group (*p* < 0.001); however, this finding likely reflects the necessity for a larger surgical access during conversion to CPB rather than an independent risk factor. When all three complication subgroups were compared, incision size was not associated with a specific complication type (*p* = 0.187).

The distribution of VSD types among patients with rhythm disturbances (both transient resolved disturbances and late cAVB) did not differ from that of the reference cohort (*p* > 0.5 for both subgroups). Perimembranous VSD, the most common defect type in the reference group (74% of all VSD types), was also predominant among patients with transient rhythm disturbances (subgroup A, 5/7 patients) and late cAVB (subgroup B, 8/10 patients). In contrast, VSD type distribution differed significantly in patients requiring conversion to CPB (subgroup C) compared with the reference cohort, with a higher prevalence of inlet VSD, absence of the aortic rim, and recanalized defects (4/8 patients in total; *p* = 0.003). Interestingly, all patients in subgroup A were treated using LifeTech occluders; therefore, occluder manufacturer was significantly associated with the incidence of transient rhythm disturbances (*p* = 0.018 vs. reference cohort). In contrast, LifeTech and Lepu devices were distributed almost equally among complication-free patients. Occluder manufacturer was not associated with late cAVB or conversion to CPB (*p* > 0.5 vs. reference cohort). Importantly, late cAVB was significantly associated with occluder configuration: this complication occurred more frequently in patients treated with eccentric occluders (*p* = 0.006 vs. reference cohort). However, occluder type was not associated with transient rhythm disturbances or conversion to CPB (*p* > 0.117 for both subgroups vs. reference cohort). Although eccentric occluders were also used more frequently among patients requiring conversion to CPB (50% of patients in subgroup C), no significant difference was identified between subgroup C and subgroups A or B (*p* = 0.110).

A larger occluder size was associated with an increased risk of late cAVB (subgroup B; *p* = 0.007 vs. reference cohort), but not with the risk of conversion to CPB (subgroup C; *p* = 0.055 vs. reference cohort). The latter association approached, but did not reach, statistical significance. Larger VSD size was significantly more frequent among patients with late cAVB (subgroup B; *p* = 0.002 vs. reference cohort) and among patients requiring conversion to CPB (subgroup C; *p* < 0.001 vs. reference cohort), suggesting a potential contribution of defect size to complication risk. Finally, in patients with late cAVB, this complication was also associated with at least one episode of viral and/or bacterial infection occurring between hybrid VSD correction and the onset of cAVB.

To validate these findings, ANOVA analysis (including main effects and factorial interactions) was performed to determine whether the identified parameters significantly influenced complication risk. In subgroup A (patients with transient rhythm disturbances), neither age, VSD size, nor anesthesia duration, either individually or in combination, demonstrated a significant effect on complication incidence (*p* > 0.74 for all parameters). These parameters also did not independently affect the risk of late cAVB (subgroup B). However, the combined effect of age and VSD size showed a borderline association with late cAVB risk (*p* = 0.06), with higher risk observed in patients older than 5 years with VSDs larger than 8 mm. Furthermore, in these older patients, the risk of late cAVB was significantly increased when anesthesia duration exceeded 2 h (*p* = 0.021). Patients in subgroup C (conversion to CPB) demonstrated a significantly higher risk of conversion when they were aged 1–5 years, had VSDs larger than 8 mm, or required anesthesia lasting more than 2 h (main effects; *p* < 0.001 for all parameters). Correspondingly, the combined effects of moderate age (1–5 years) with large VSD size (>8 mm) or prolonged anesthesia duration (>2 h) significantly increased the likelihood of CPB conversion (all interaction effects, *p* < 0.001). Similarly, the combination of large VSD size and anesthesia duration >2 h markedly increased the probability of conversion to CPB (*p* < 0.001).

Importantly, VSD type in combination with defect size was not a significant interaction factor influencing the incidence of transient rhythm disturbances or late cAVB (*p* > 0.6 for both subgroups). In contrast, in subgroup C, the presence of an inlet VSD, either alone or combined with a larger defect size, was significantly associated with an increased likelihood of conversion to CPB (*p* < 0.001). Occluder type alone or in combination with VSD size was not associated with an increased risk of transient or late rhythm disturbances (*p* > 0.18 for both main and interaction effects). However, in subgroup C, the use of an eccentric occluder significantly increased the risk of conversion to CPB, both independently and in combination with larger VSD size (*p* < 0.001 for both analyses).

## 4 Discussion

Open-heart surgery requires the use of extracorporeal circulation, also known as cardiopulmonary bypass (CPB), because cardiac arrest is necessary to allow intracardiac surgical manipulation. Conventional surgical repair remains the gold standard for VSD correction and has been the most widely used approach for several decades (23). However, pediatric patients, particularly infants and toddlers, represent a highly vulnerable population undergoing CPB-associated procedures. Potential risks include systemic inflammatory responses and thromboembolic complications resulting from extracorporeal blood circulation, ischemic neurological injury due to reduced cerebral oxygen delivery during on-pump procedures, and adverse neurocognitive outcomes associated with prolonged anesthesia and hemodilution (9, 24–26). For example, in very young infants, the incidence of postoperative cerebral injury, ranging from transient abnormalities to severe neurological complications, may reach 50% (27). Notably, conventional VSD repair under CPB is generally performed with arterial perfusion pressures approximately half of normal physiological levels and with hemodilution, both of which represent additional risk factors for encephalopathy and neurological complications. Acute kidney injury following cardiac surgery with CPB is another important postoperative complication, occurring in approximately 30%–40% of pediatric patients (28). In addition, prolonged CPB duration has been associated with increased myocardial cellular injury, as demonstrated by elevated plasma levels of cardiac troponin T (29), cardiomyocyte-specific cell-free DNA (30), and ionized calcium concentrations (31) in children undergoing open-heart surgery. This molecular evidence of increased cardiomyocyte death may contribute to impaired postoperative left ventricular ejection fraction (LVEF) in patients undergoing CPB compared with CPB-free procedures. Indeed, CPB duration has been identified as an independent risk factor for low cardiac output syndrome, which contributes to postoperative mortality in pediatric patients (32). Additional concerns associated with conventional surgery include postoperative pain caused by full median sternotomy and subsequent scar formation (33). Furthermore, open-heart VSD repair with CPB is associated with substantial surgical trauma, increased risk of wound complications, prolonged recovery, and less favorable cosmetic outcomes (34). Finally, conventional VSD repair is technically demanding and requires close coordination among multidisciplinary teams, thereby increasing procedural complexity and healthcare resource utilization.

To reduce these disadvantages, minimally invasive approaches, including mini-sternotomy and mini-thoracotomy, have been introduced. For example, a recent study demonstrated that modified right vertical infra-axillary thoracotomy (MRVIAT) resulted in significantly shorter CPB duration and reduced ICU and hospital stays compared with conventional median sternotomy (35). However, the median postoperative ICU stay reported in these MRVIAT patients remained substantially longer than that observed in our cohort. Another study evaluated MRVIAT and right submammary thoracotomy in pediatric patients with perimembranous VSD and demonstrated comparable outcomes between these approaches, with significantly shorter ICU stays compared with conventional surgery (36). Interestingly, the ICU and hospital stay durations reported in that study were comparable with our results, whereas the duration of mechanical ventilation was considerably longer than that observed in our cohort. Nevertheless, a substantial proportion of studies involving both very young infants (∼3 months of age) and older children have failed to demonstrate significant differences in postoperative recovery parameters between conventional surgery and mini-thoracic approaches (37, 38). Overall, although these techniques may reduce postoperative recovery time, require smaller incisions, and improve cosmetic outcomes, they still require CPB and therefore remain associated with longer procedures and recovery periods compared with CPB-free approaches.

In this context, transthoracic device closure, commonly referred to as hybrid or periventricular VSD repair, represents a promising alternative to conventional surgical VSD closure in patients with favorable anatomical characteristics. This approach combines direct surgical access with interventional device deployment under echocardiographic guidance and has demonstrated particular effectiveness in low-weight infants and patients with muscular VSDs (16, 39). Although the term “hybrid” may include procedures performed with limited CPB assistance, contemporary clinical practice and recent literature predominantly refer to CPB-free hybrid interventions (40). In our series, the vast majority of patients underwent successful VSD closure without CPB, with only a small proportion requiring intraoperative conversion to conventional surgical repair. Only a few cases of tricuspid regurgitation occurred during the hybrid procedure, and these patients were immediately converted to CPB. Among patients successfully treated with the hybrid approach, no delayed progression of tricuspid valve insufficiency was observed during follow-up. Therefore, our findings suggest that hybrid VSD repair does not carry a substantial risk of clinically significant tricuspid regurgitation.

Furthermore, none of the patients required reoperation, as no patient developed a hemodynamically significant residual VSD, despite the presence of relatively large residual defects in some cases (e.g., ≥4 mm in seven patients). All patients with residual shunts demonstrated preserved LVEF (≥60%). It should be noted that, in the present study, any flow detected by color Doppler echocardiography was classified as a residual shunt. However, some of these findings may represent false-positive detections, such as localized non-laminar flow adjacent to the occluder edge without true trans-septal shunting. The overall incidence of hemodynamically insignificant residual shunts (11.4% in our study) and their mean size were comparable with previously published data from studies involving infants and children, in which reoperation was not required (41). Furthermore, our clinical experience indicates that most residual shunts undergo spontaneous closure within 36–72 months, consistent with numerous previously reported observations.

Appropriate patient selection remains a critical determinant of procedural success. Conventional surgical closure is generally recommended for large perimembranous VSDs, whereas the hybrid approach is particularly advantageous in small infants with large muscular defects (42). Although hybrid repair has been successfully applied in patients with complex anatomy, single-ventricle physiology, left heart hypoplasia, and high-risk or reoperative settings (43–47), in the present study we selected patients with favorable VSD anatomy for this technique, specifically those with an aortic rim of at least 2 mm. Overall, reported procedural success rates for hybrid VSD repair are consistently high, frequently approaching 97% (48). Earlier multicenter experience also demonstrated successful hybrid device placement in approximately 88.5% of cases, particularly among patients with muscular VSDs (49).

Compared with conventional surgery and transcatheter closure, hybrid VSD repair is generally associated with shorter operative duration and reduced overall anesthesia time, potentially decreasing anesthesia-related complications (39). Unlike transcatheter approaches, the hybrid technique may also reduce the risk of vascular injury and valve damage associated with catheter manipulation through major vessels. Nevertheless, despite these advantages, large-scale studies with extended follow-up remain limited, emphasizing the need for further institutional experience and long-term outcome analyses.

In addition to procedural feasibility, increasing attention has been focused on the long-term outcomes of hybrid VSD closure, particularly with regard to residual shunts, device stability, and late conduction disturbances. Available mid- and long-term follow-up data indicate that complete closure rates after hybrid repair remain high, while the incidence of complete atrioventricular block (cAVB) and clinically significant device-related complications appears to be comparable to or lower than that reported after conventional surgical or transcatheter closure, particularly in patients with muscular VSDs (16, 43, 49). Importantly, avoidance of CPB may provide neurodevelopmental advantages in neonates and young infants, as early-life exposure to extracorporeal circulation and prolonged anesthesia has been associated with adverse cognitive and behavioral outcomes (50–52). Furthermore, because the hybrid approach is associated with shorter ICU stays, it may be expected that patients undergoing hybrid repair would demonstrate lower circulating biomarkers of myocardial injury compared with patients undergoing CPB-based surgery; however, such comparative data are currently lacking.

Taken together, these findings support the concept that the hybrid periventricular approach may serve as a valuable complementary strategy to conventional surgery and may replace it in selected patients within a tailored, anatomy-based treatment algorithm. Nevertheless, further multicenter studies with standardized follow-up protocols are required to more accurately define its long-term efficacy and safety (53, 54). Meanwhile, conventional open-heart surgery with CPB remains the predominant surgical approach in pediatric and adult cardiac surgery worldwide due to its broad availability, extensive clinical experience, and relatively lower equipment requirements. Similar to many cardiac surgery centers worldwide, our institution continues to perform both conventional open-heart surgery and hybrid VSD repair. Since introducing the hybrid technique in 2016, we have routinely applied both approaches, with approximately comparable numbers of procedures performed (∼1,100 conventional repairs vs. ∼800 hybrid repairs). Conventional surgery is preferentially selected when the VSD diameter exceeds 10 mm and/or when unfavorable anatomical features are present, such as absence of the aortic rim with prolapse of the aortic valve septal leaflet into the defect. In addition, concomitant severe aortic insufficiency requires correction under CPB. Inlet VSDs, in which the defect margin is located close to the tricuspid valve, also represent an important indication for conventional surgery because occluder deployment in such cases may increase the risk of atrioventricular block or tricuspid valve dysfunction. Conversely, we prefer the hybrid approach in patients with suitable VSD size, favorable anatomy, and appropriate body weight, as this strategy allows avoidance of CPB-related risks, reduction of intraoperative and postoperative morbidity associated with smaller surgical access, improved cosmetic outcomes, and shorter rehabilitation periods.

It should be acknowledged that 35 children (4.7% of the initial cohort) were lost to follow-up; therefore, our findings may be partially affected by incomplete long-term observation. However, based on a review of available medical records performed in 2024 through authorized and approved access, none of these patients were documented as deceased. Based on our extensive clinical experience with hybrid VSD repair in children of different ages and our comprehensive analysis of early and long-term outcomes, we conclude that early postoperative transient rhythm disturbances are not associated with patient age, VSD size, VSD type, occluder type, or anesthesia duration. However, late severe rhythm disturbances presenting as cAVB occurred in a small proportion of patients (1.3%) undergoing hybrid repair, with onset ranging from two weeks to 3.5 years after surgery. Analysis of potential risk factors demonstrated that the combination of older age (>5 years) and larger VSD size (>8 mm) represented important predictors of late cAVB. Notably, VSD size alone was not an independent risk factor. Another important finding was that inlet VSD type, larger VSD size, and particularly the combination of these two factors were significantly associated with the need for conversion to CPB. These findings emphasize that specific anatomical and clinical characteristics should be considered when evaluating perioperative and postoperative risks in selected patients. Consistent with our institutional experience, the presence of an inlet VSD appears to be associated with an increased risk of conduction disturbances; therefore, we preferentially select conventional CPB-based repair for these patients.

Complete atrioventricular block (cAVB) after cardiac surgery for congenital heart defects (not limited to VSD repair) may occur in approximately 1 out of 30–35 children, according to a large registry-based study including approximately 16,000 pediatric patients from 25 hospitals (55). Importantly, these early postoperative conduction disturbances resolve spontaneously in approximately 94% of affected patients within 10 days. The mechanism and clinical course of conduction disturbances after occluder implantation differ substantially from those observed after conventional VSD patch closure, primarily due to the specific geometry and mechanical properties of the occluder device. In addition to early rhythm disturbances associated with direct surgical manipulation of the myocardium, the occluder edges may exert mechanical pressure on the surrounding ventricular septal tissue and potentially damage adjacent conduction pathways. Importantly, the adverse effects of mechanical compression may not become apparent immediately after device implantation. Instead, progressive interaction between the implanted occluder and the surrounding myocardium during cardiac growth may result in delayed tissue injury and conduction system impairment. Therefore, patients undergoing hybrid VSD repair remain at risk of delayed (rather than immediate postoperative) atrioventricular block. For example, in our cohort, one patient developed AV block 3.5 years after surgery, while another developed this complication 2 years postoperatively. These observations emphasize the necessity of long-term surveillance after hybrid VSD repair. Since this potential complication remains insufficiently investigated, our study contributes important additional data to the limited evidence currently available. Based on our findings, we propose updating clinical follow-up recommendations for patients undergoing hybrid VSD repair:

- Enhanced long-term monitoring is required. We strongly recommend electrocardiographic (ECG) evaluation at least twice annually for a minimum of 10 years after hybrid VSD closure in all patients. Lifelong surveillance should be considered in patients with additional risk factors, including close proximity of the occluder to the conduction system, particularly when eccentric devices are used, and the presence of early postoperative ECG abnormalities.
- Patients and their parents should receive appropriate counseling regarding symptoms potentially associated with bradyarrhythmias, including syncope, dizziness, weakness, and reduced exercise tolerance, and should be instructed to seek immediate medical evaluation if such symptoms occur.
- The informed consent process should include a specific warning regarding the possibility of delayed complete atrioventricular block, which may develop several years after the procedure.

**Figure 4:**
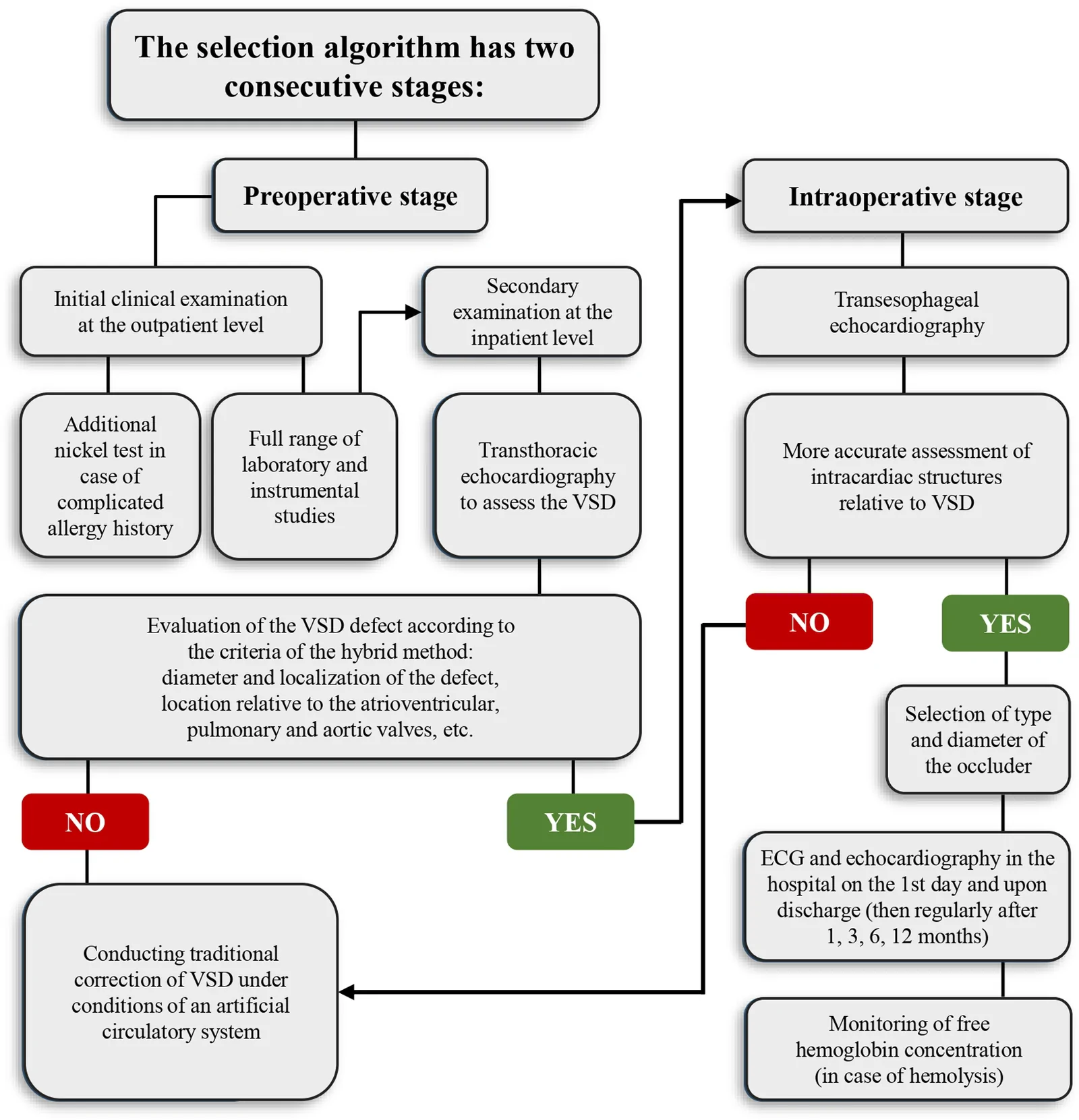
Algorithm for the selection of pediatric patients for transventricular VSD correction. The algorithm consists of two consecutive stages: preoperative and intraoperative evaluation. The flowchart illustrates the decision-making pathway for conversion from the hybrid technique to conventional open-heart surgery in specific situations, particularly when accurate assessment of VSD-related intracardiac anatomical structures is not feasible.

Based on our institutional experience and previously published data, we developed a clinical algorithm for patient selection for hybrid periventricular VSD repair. The algorithm is presented in Figure 4 and consists of two sequential stages: preoperative evaluation and intraoperative decision-making. The preoperative stage includes an initial outpatient assessment comprising a comprehensive set of laboratory and instrumental investigations, with additional nickel allergy testing in patients with a relevant allergy history. If required, a second inpatient evaluation is performed, including transthoracic echocardiography to assess VSD characteristics according to predefined criteria for hybrid repair. If the VSD anatomy does not meet the criteria for hybrid correction, the patient undergoes conventional surgical VSD repair with CPB. If the VSD meets the criteria for hybrid repair, the second stage of the algorithm—the intraoperative stage—is initiated. During this stage, transesophageal echocardiography is performed to provide a more detailed assessment of intracardiac anatomy and the relationship between the defect and surrounding structures. If hybrid correction remains appropriate after intraoperative assessment, the optimal occluder type and size are selected, followed by device implantation. The final component of the intraoperative stage involves postoperative in-hospital monitoring, including ECG and echocardiographic evaluation on the first postoperative day and before hospital discharge. Subsequent follow-up examinations are scheduled at 1, 3, 6, and 12 months after the procedure, followed by long-term surveillance. Free hemoglobin concentration is also monitored when clinically indicated to detect possible hemolysis.

Finally, we would like to briefly address the economic aspects of the surgical approaches, particularly direct procedural costs. Our unpublished data obtained from very young children (<1 year of age) indicate that hospital stay costs are approximately 1.5-fold higher in patients undergoing CPB-based surgery, with ICU-related costs also being substantially greater. Conversely, direct surgical costs, including the price of the occluder device, are approximately twofold higher in the hybrid surgery group compared with the CPB group. Overall, the total cost of hybrid surgery was approximately 1.6-fold higher than that of CPB-based surgery in this population of very young children. However, we observed an important trend toward a gradual reduction in the overall cost of hybrid procedures over the past decade (based on our institutional dataset from 2016 to 2024), with the total costs of hybrid and CPB-based surgery becoming nearly comparable in the most recent year of analysis. In contrast, the overall costs of CPB-based surgery remained relatively stable over the same period. The primary factor contributing to the reduction in hybrid surgery costs is likely the decreasing cost of occluder devices. Accordingly, further expansion of hybrid VSD repair may be expected as its economic feasibility and clinical adoption continue to improve.

## 5 Conclusion

**Figure 5:**
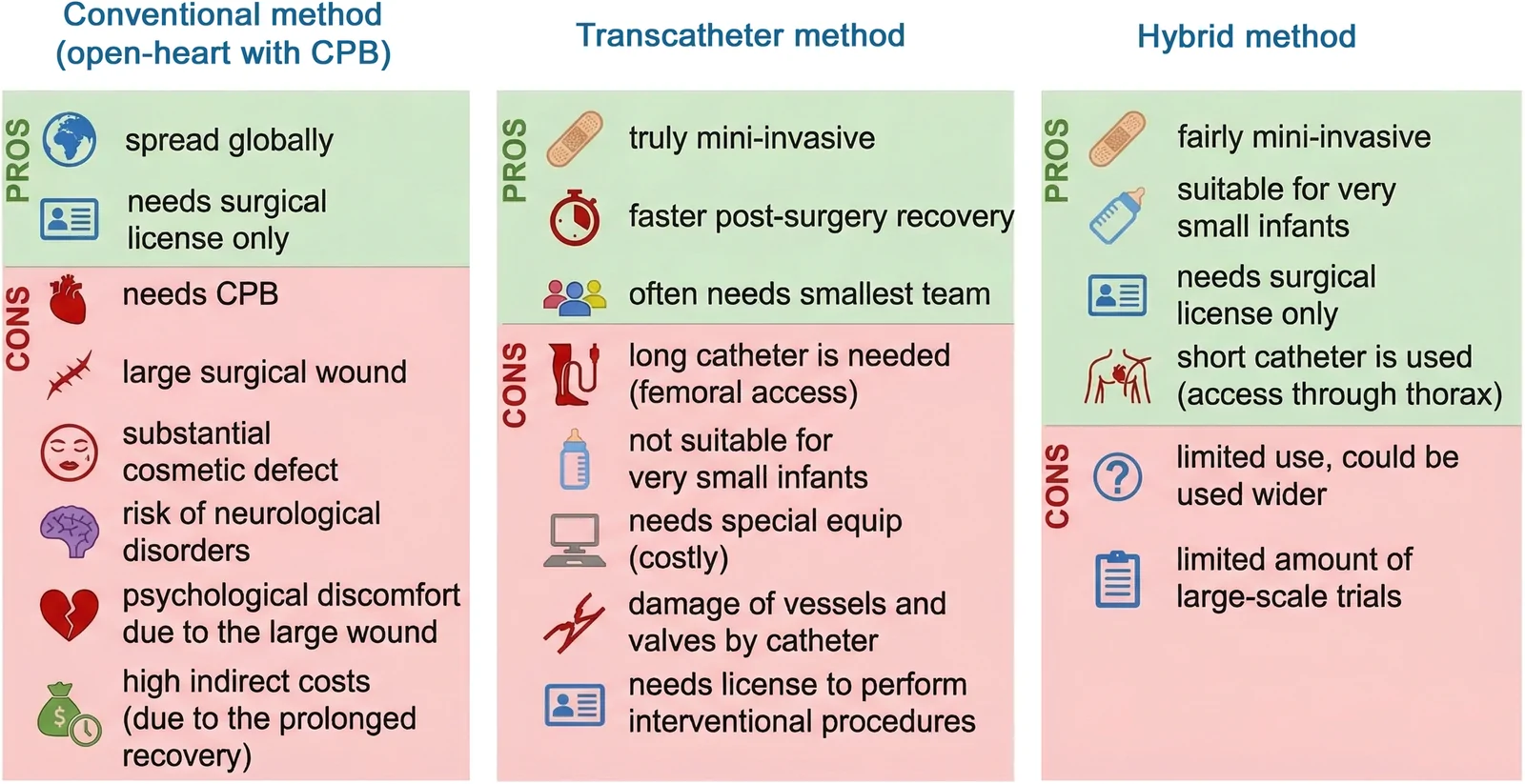
Schematic comparison of the advantages (pros, green area) and limitations (cons, red area) of three major approaches for VSD closure: conventional open-heart surgery (left column), transcatheter device closure (middle column), and hybrid periventricular repair (right column).

The results of this study demonstrate that the hybrid periventricular technique for VSD correction is highly effective and safe in children when applied according to clearly defined indications and in appropriately selected patients. The short ICU and hospital length of stay observed in our cohort supports the minimally invasive nature of this approach and its potential to reduce rehabilitation time. The incidence of long-term complications was relatively low. Hybrid VSD closure has several distinctive advantages that make it a promising alternative to conventional open-heart surgery and, in selected cases, to transcatheter approaches. Figure 5 summarizes the key characteristics of all three treatment strategies, highlighting their respective advantages and limitations. It should be noted that the current lack of large-scale studies evaluating the hybrid technique represents a temporary limitation that requires further investigation through multicenter studies and extended follow-up.

## Data Availability

The raw data supporting the conclusions of this article will be made available by the authors, without undue reservation.
